# Determination of critical diameters for intrinsic carrier diffusion-length of GaN nanorods with cryo-scanning near-field optical microscopy

**DOI:** 10.1038/srep21482

**Published:** 2016-02-15

**Authors:** Y. T. Chen, K. F. Karlsson, J. Birch, P. O. Holtz

**Affiliations:** 1 Department of Physics, Chemistry and Biology (IFM), Linköping University, SE-58183 Linköping, Sweden

## Abstract

Direct measurements of carrier diffusion in GaN nanorods with a designed InGaN/GaN layer-in-a-wire structure by scanning near-field optical microscopy (SNOM) were performed at liquid-helium temperatures of 10 K. Without an applied voltage, intrinsic diffusion lengths of photo-excited carriers were measured as the diameters of the nanorods differ from 50 to 800 nm. The critical diameter of nanorods for carrier diffusion is concluded as 170 nm with a statistical approach. Photoluminescence spectra were acquired for different positions of the SNOM tip on the nanorod, corresponding to the origins of the well-defined luminescence peaks, each being related to recombination-centers. The phenomenon originated from surface oxide by direct comparison of two nanorods with similar diameters in a single map has been observed and investigated.

Semiconductor nanorods/nanowires are considered as building blocks in advanced devices, e.g. for energy harvesting/transformation/conservation^1^, bio-molecular and gas sensing^2^, optoelectronic detectors/emitters^3^,^4^, spintronics, and nano-scale electronics^5^. Diffusion of charge carriers in the nanorods is a fundamental property of major concern for applications mentioned above. For example, an improved performance has been monitored in GaAsP and GaAs solar cells with a 1-D geometry owing to the increased minority carrier diffusion length due to the surface passivation^6^,^7^. Cathodoluminescence (CL), as an option in both SEM and TEM, has been employed to measure the carrier diffusion in samples with a p-n junction or an embedded single quantum well^8^,^9^,^10^. Detection of hole accumulations have been successfully reported in p-type Si/Ge quantum dot/wire^11^,^12^. However, there are essential considerations about the CL results, such as charging effects and damages of the rod due to the bombardments of high-energy electrons, local heating of the sample surface, which in turn will broaden the observed CL emission peak. On the other hand, electrical methods performed on single nanorods including measurements of photoconductivity, photocurrent, and dark currents provide indirect evidences for diffusion-length estimation. The challenges mainly come from the need of applied external voltages in these measurements since the signals of electrical current will become undetectable with applied voltage less than 1 to 0.1 volt^13^. As a consequence, the carrier diffusion along the surface is difficult to monitor by state-of-the-art techniques.

As a novel route, scanning near-field optical microscopy (SNOM/NSOM) has been developed during the last decades. SNOM, with its ability operating with evanescent waves, opens a unique possibility to detect features smaller than the laser diffraction limit^14^,^15^,^16^. In this way, SNOM offers an exclusive approach to gain optical information characterized by a high spatial and spectral resolution, combined with a simultaneous high flexibility to guide the signal to different setups for various kinds of optical analysis. Reports using SNOM to detect thin films or single quantum wells^17^,^18^ for the distribution of carrier densities can be found. Carrier/exciton diffusion with different crystalline planes and different modes of SNOM operations with single/dual probe-setups has been investigated in the room temperature^19^.

The main drawback of the SNOM is the low scanning rate and the weak signal level compared to conventional optical spectroscopy. Accordingly, the stability of the stage and the SNOM tip is crucial for this kind of carrier diffusion experiments, especially at low temperatures. Due to the limited UV transmission of optical fibers, most SNOM related studies have been performed in the infrared regime and at room temperature. Only very few reports at low temperature (36 K) can be found working in the UV region^20^ for quantum wells.

GaN nanorods exhibit promising properties in terms of an ultra-high photoconductive gain^21^,^22^. Owing to the surface band bending induced by the Franz-Keldysh effect and the pinning of the Fermi level at the sidewall^23^, a long electron-hole recombination lifetime and consequently an ultra-high photoconductive gain has been observed^21^. Although there is a manifold of ways to indirectly measure the carrier distribution and diffusion by means of an applied voltage across the nanorod, direct measurements have not been reported up to date. The carrier behavior and diffusion length have to be monitored directly. Further, the impact of the surface on the electronic band structure has to be elucidated in a direct way without an applied voltage.

In this study, measurements of carrier diffusion in single InGaN/GaN layer-in-a-wire structures with SNOM were performed at low temperatures (10 K). Without an applied voltage, diffusion lengths of photo-excited carriers were measured directly as the diameters of the nanorods differ from 50 to 800 nm. Recombination-centers were investigated for different positions of the SNOM tip on the rods. The effect of surface-oxide can be clearly observed as well.

## Results and Discussion

**Figure 1:**
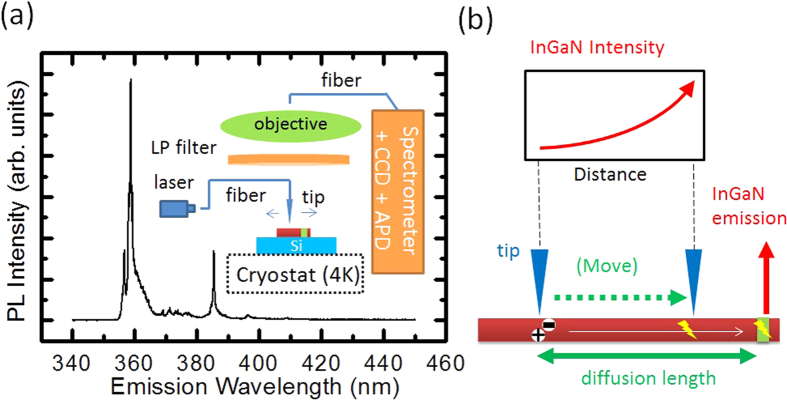
SNOM experiment. (**a**) μ-PL spectrum (4 K) observed from an ensemble of nanorods with a single InGaN insertion layer. The inset shows the setup of the SNOM experiment. (**b**) Illustration of the SNOM experiment for the measurements of the carrier diffusion profile.

An illustration of the experimental setup for SNOM is shown in the inset of Fig. 1(a). Detailed description can be found in the method section. The reflection mode of near-field excitation and far-field collection has been employed. Prior to the SNOM experiments, low temperature (10 K) micro-photoluminescence spectroscopy has been performed on an ensemble of wires dispersed on a piece of silicon substrate. The near band-edge emission (NBE) appearing around 359 nm (see Fig. 1(a)) is well matching the reported emission of the near bandgap excitons in the MBE-grown GaN nanowires^24^. The emission from the InGaN insertion layer at around 385 nm with a smaller FWHM than the NBE, but still with a high emission intensity. The evanescent wave of the UV excitation from the aperture can excite the nanorod body within a defined area with a diameter of 50–200 nm, as corresponding to the size of the aperture. Electrons and holes can be generated and diffuse in the wurtzite GaN nanorod along the (0001) axial rod direction, and recombine in the rod body (as a NBE), or become captured in the lower-band-gap InGaN layer if the tip is close enough. Since an optical 364 nm long-pass filter, blocking the NBE signal, is employed, only the emission of the InGaN insertion layer can be detected. In addition, a 532 nm short-pass filter is applied in order to suppress other background emissions. An exponential increase of the emission intensity is expected, when the SNOM tip is moved towards the InGaN layer, as shown in Fig. 1(b).

**Figure 2:**
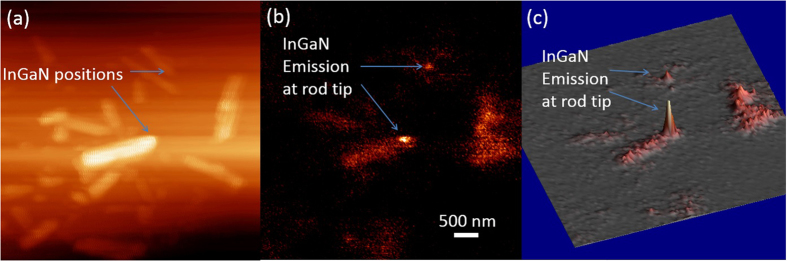
High PL intensity from the InGaN layer can be observed clearly in the SNOM map. The map of (**a**) the height and (**b**) the photoluminescence obtained simultaneously in a single SNOM scan at 10 K. (**c**) The 3D version of (**b**).

The outcome of the SNOM scanning on single rods without an optical filter applied is shown in Fig. 2. The morphology and luminescence image is obtained simultaneously, as shown in Fig. 2(a,b), respectively. Fig. 2(a) is a conventional height map with the intensity calculated from the amplitude of the oscillating cantilever. For Fig. 2(b), every pixel represents a panchromatic PL intensity, when the tip is placed at that particular position, excited by the evanescent wave through a well-defined small aperture at the tip. A distribution of rod diameters is observed, from 50 nm up to 800 nm. In the PL map, the InGaN insertion layer is observed at the tip part of the rod and has a much higher intensity than the rod body (as shown in Fig. 2(b,c)). This fact can be attributed to the lower band gap of the InGaN insertion layer, which accordingly will capture excited electrons and holes. The In composition fluctuates in the range between 5 and 10%, which corresponds to an indium composition with a high luminous efficiency of the InGaN material. Since the InGaN emission is well separated in energy from the GaN emission, a long-pass optical filter of 364 nm efficiently blocks the emission of GaN. There is a significant correlation between rods exhibiting decent luminous efficiencies and rods having larger diameters (>200 nm) (see Fig. 2(a,b)) for a population of about 10 nanorods. The nanorods with an adequate PL intensity for both the GaN and the InGaN appearing in the center of the image are selected in this report for the diameter-dependent statistics later on. Simultaneously, a much smaller nanorod with the InGaN emission at the tip can be detected as well (in the upper part of this figure), although the emission from the rod body is too weak to be detectable. The detectable PL intensity is heavily dependent on the nanorod diameter.

**Figure 3:**
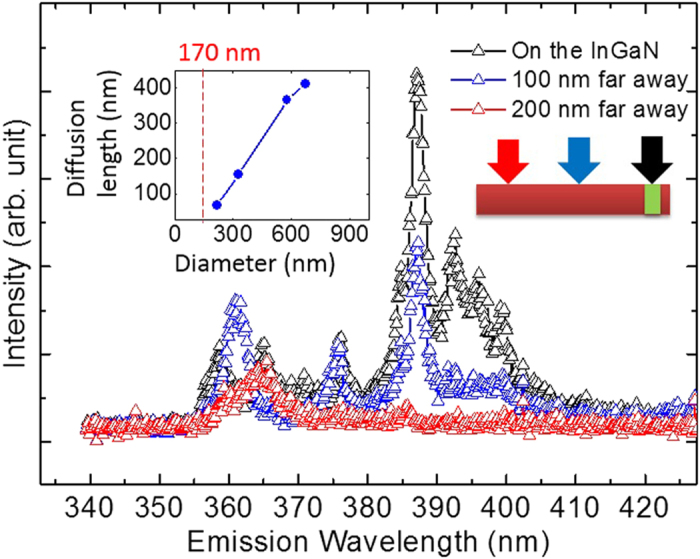
Luminescence spectra measured at 10 K for different tip positions on the nanorod. The distances between the SNOM tip and the InGaN layer are i) 200 nm (red), ii) 100 nm (blue) and iii) 0 nm (black), respectively. The inset shows the linear dependence of the diffusion lengths on the rod-diameter as the outcome of the experimental survey. From an extrapolation, the photo-excited holes in the rods with diameters smaller than 170 nm will exhibit non-detectable diffusion.

An advantage of SNOM is the possibility to use a conventional spectrometer to analyze the emission with a high spectral resolution at any location on the rod overcoming the diffraction limit. Such spectra obtained via the SNOM tip, located at different positions, are shown in Fig. 3. The tip is positioned at different spots: i) on the InGaN layer, ii) 100 nm away from the InGaN layer, and iii) 200 nm away from the InGaN layer. When the tip is located at the InGaN layer, the intensities of the InGaN emission peaks (between 370 and 410 nm) are dominating over the GaN NBE emissions, but gradually decreases as expected, when the tip is moved away. The observed multiple peaks indicate the existence of multiple localization-centres of excitons in the InGaN layer. Similar recognitions made in typical InGaN thin films or quantum wells/dots have been attributed to localized region of higher indium-content or atomic In-N zigzag chains in the structure^25^. Even if the intensities of InGaN peaks successively degrade as the tip is moved 200 nm away from the InGaN, the peaks at higher energies (around 387 nm) are still detectable, while the peaks at longer wavelengths have been quenched since they have an overall lower intensity. The 387 nm and the 390–400 nm peaks follow each other proportionally. The longer distance between the excitation source and the target InGaN layer will result in a decreasing number of carriers diffusing and captured into the emitting InGaN layer. This observation implies that the localized states related to the 387 nm peaks are either closer to the base of the nanorod compared to other related localized states appearing at longer wavelengths, so the carriers diffusing into the InGaN layer will be trapped there and recombine, or they have higher quantum efficiencies, which will still give a radiative recombination under the reduced excitation power, while the other low-efficient localized states are quenched due to a high rate of non-radiative recombination.

By applying a 364 nm long-pass optical filter to block the GaN emission, the InGaN emitter can be directly traced on the single nanorod in a PL mapping. Based on an extended statistical basis, achieved by means of multiple images of the nanorods, the relationship between the rod diameter and the diffusion length of carriers has been investigated (see the inset of Figs 3 and S1). The diffusion lengths have been evaluated from a fitting procedure by single exponential functions. A linear decrease of the diffusion length with a decreasing rod diameter is observed, with a critical threshold at 170 nm deduced as minimum rod diameter for monitoring a detectable carrier-diffusion at the surface. The trend matches with the commonly observed result in the literature with the existence of surface band bending^21^,^22^,^23^. According to the proposed model, the non-depletion region which is responsible for carrier diffusion becomes narrower when the diameter is smaller. However, our concluded critical diameter is larger than estimates on the corresponding critical diameter of around 80 nm based on electrical measurements with an applied voltage over depletion region of the nanorods^22^, since no voltage is applied in our case. In addition, reports based on cathodoluminescence experiments^9^ on MBE-grown nanorods with a p-n junction imply that the hole-diffusion length is around 165 nm at the temperature of 93 K for a rod diameter of around 200 nm, i.e. a larger value than our observed carrier diffusion length of 67 nm at 10 K for a rod with a similar diameter. However the CL characterization involves a high energy electron beam excitation, resulting in an, electron-induced damage on the surface, which can be a factor affecting the diffusion length. The existence of an electric field either in the depletion regions of the p-n junction or from the external applied voltage could also affect the measured diffusion length as well.

**Figure 4:**
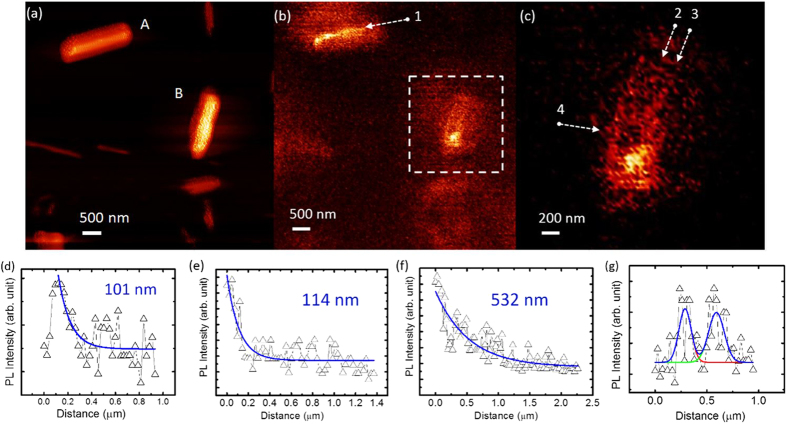
SNOM maps and spectra provided with an optical 364 nm long-pass filter applied. (**a**) Maps of the height show that several rods with different diameters ranging from 50 up to 500 nm are monitored, including a rod with flat sidewalls at the upper position (marked as A), and a rod with rough surfaces at the middle-right position (marked as B). (**b**,**c**) PL maps recorded simultaneously with (**a**). The profile was analyzed along the lines marked by 1 (from rod A), 2, 3 (from rod B), and shown separately in the figures (**d**–**f**), respectively. The diffusion lengths were calculated to be 101 nm, 114 nm, and 532 nm, respectively, from a fitting procedure by single exponential decay curves. A cross-sectional profile was taken along the line marked by 4 (rod B) in (**c**), and the result is shown in (**g**), which clearly illustrate that the PL intensities are stronger at both the left and right sidewalls and weaker at the center of the rod. The distance between the two fitted peaks is 303 nm.

In order to investigate the behavior of carriers diffusing at the rod surface, a comparison between two rods with similar diameters has been performed, as shown in Fig. 4. With the long-pass optical filter applied, the two rods scanned exhibit different signatures. The intensity in the maps represents the PL emission from the InGaN layer. The rod B with a rough sidewall surface, marked as B in Fig. 4(a), exhibits a stronger PL intensity at the edge of the rod than at the center, in contrast to the upper rod with the same diameter, as shown in Fig. 4(b,c). The upper rod (marked as A) with a well-defined shape and flat sidewalls exhibits a carrier diffusion length of 101 nm (Fig. 4(d)), while the rod at the middle-right (marked as B) position has a rougher surface and a unique SNOM map. The PL signal of the rod B exhibits a stronger InGaN emission, for the SNOM tip located at the edge of the rod versus a tip location at the center (as shown in Fig. 4(c)). Since any 2D microscopy refers to the projection of the image of a 3D object, the center here refers to when the tip is put on the rod center under the illumination of the laser excitation, but still the in-plane surface is also illuminated. However, the surface/body ratio of illuminated volume is much smaller than for the case, when the tip is put on the edge of the rod. For measurements along the edge and the center, respectively (shown by arrows 2 and 3 in Fig. 4(c)), the diffusion length extracted from the intensity decay at the edge of the rod is estimated to be 530 nm, and is about five times longer than the diffusion length at the center (110 nm). From the cross-sectional profile shown in Fig. 4(c,g), shown by arrow 4, both the left and right sides of the rod exhibit a strong PL intensity from the InGaN layer. The luminescence intensities are similar on both sides, but the width is larger for the right side than the left side, due to the fact that the major InGaN emission-center is located close to the right side of the rod. It is found that the roughness of the surface plays an important role for the difference in PL intensities between the edge and the center.

**Figure 5:**
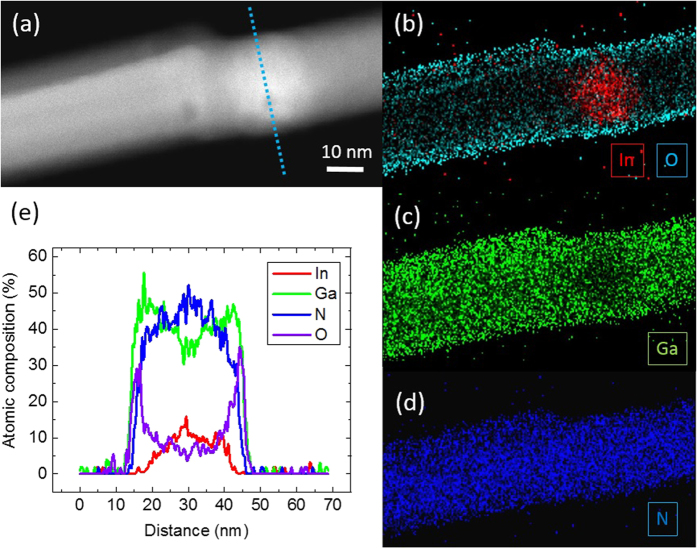
STEM-EDX images on a nanorod. (**a**) HAADF image indicates the diameter of the rod is around 30 nm. The compositional map of indium, oxygen, gallium, and nitrogen are obtained, as shown in (**b**–**d**), respectively. (**e**) A line profile is obtained at the location of InGaN layer on the rod, as indicated as the blue line in (**a**). At the side surfaces of the rod, the regions of high oxygen content are found with thickness around 7 nm. Regions with 2 nm thickness contain only gallium and oxygen are observed as well with the absence of nitrogen, which indicate the existence of gallium oxide.

In order to explain the center-sidewall difference in Fig. 4, scanning transmission microscopy with energy-dispersive X-ray spectroscopy (STEM-EDX) is performed to identify the chemical composition of the nanorod, as shown in Fig. 5. On the sidewall of the rod, the oxygen content is found to be much higher than in the center part, indicating the existence of oxide layer, as shown in Fig. 5(b). Meanwhile, the nitrogen content is almost zero at the surface, as shown with the cross-sectional profile in Fig. 5(e). The indium content in the InGaN layer is varied, and the trend is complementary for gallium content. The existence of multiple localized high indium-regions matches with the multiple PL peaks observed in the spectrum shown in Fig. 3.

Nanorods with rough surface are commonly expected to have more dangling bonds and defects at the surface, which provide more nucleation sites for the growth of the oxidation layer. Therefore, thicker oxide layers can be expected to form compared to the smooth counterpart. The formatted gallium oxide at the surface of the rod has a refractive index known to be around 1.8 26 with respect to the UV illumination. Meanwhile, the band gap of gallium oxide is around 4.8 eV, which is higher than the laser energy and allows the laser beam to travel along the sidewall without an absorption or a creation of photo-excited carriers. Therefore, with a thick surface oxide on the sidewall of the rough rod, an injected laser beam can be partly absorpted by the GaN body, and be partly scattered and traveling in the surface gallium oxide along the axial direction, and reach the InGaN layer. Since oxide exist on the side surface of all rods with varied thickness depending on the surface flatness, and the body-surface difference shown in Fig. 4 cannot be observed in normal rods with flat and thin oxide surface, the dominating factor to cause the body-surface difference is not the carrier diffusions related to the oxide. Therefore, the preferable explanation is that the laser goes along the side wall in the thick oxide, and then reaches the InGaN much easier than the flat counterpart, since the thickness of the oxide on flat sidewall is only 2 to 7 nm, as shown in Fig. 5. From the perspective of the investigations of carrier diffusion lengths, one therefore has to be careful and to take the surface oxide and surface roughness into consideration.

The carrier diffusion in GaN nanorods without a voltage bias has been investigated by means of SNOM at 10 K. With an InGaN insertion layer at the top emitting at different wavelengths, the diffusion lengths of the photo-excited carriers have been measured directly. Critical diameter of the nanorod for the carrier diffusion is concluded to be 170 nm. Thanks to the high resolution of SNOM, the effect of gallium oxides formed at the surface can be observed as well, which is confirmed by STEM-EDX investigations. This phenomenon is observed to be more prominent on the rods with rough sidewall, by a direct comparison of two nanorods in a same SNOM map.

## Methods

### Crystal growth

The InGaN-layer/GaN-rod structures are grown by molecular beam epitaxy on top of a clean silicon (111) substrate. After the growth of the GaN nanorods, a single 10 nm thick InGaN insertion layer followed by a thin GaN capping layer were deposited. The InGaN layer serves as an optical landmark, which emits photons at wavelengths different from the GaN rod body. The fabrication details, structural and optical properties of this structure can be found in our previous work^4^,^27^.

### SNOM

Cryo-SNOM, operated at 10 K and in the reflection mode, has been employed without any applied external voltage for investigations of the fundamental properties of the photo-generated carriers diffusing in the nanorod. The SNOM setup (Nanonics CryoView) equipped with a cryostat with an open-loop, recycled liquid-helium setup in a chamber with electronics of scanning probe microscope (SPM) characterized by a lateral precision of 4 nm. The morphology and phase information is then collected through the oscillating cantilever of the SPM with a frequency around 35 kHz, different slightly for each SNOM tip. A tip, made of an optical fiber coated with gold and chromium at the tip part, which defines the aperture and the resolution, is responsible for the scanning of the nanorod in the tapping mode. The size of the opening used in the experiment is 50 nm. The evanescent wave propagates from the aperture and generates carriers in the nanorod within the area defined by the aperture. The emission is collected from a long working-distance optical reflective objective (20X) to increase the UV transmission efficiency, and with optional an optical filter (Semrock 364 nm long-pass) blocking the emission of GaN. The emission signal is guided via an optical fiber in the far field into an avalanche photodiode (APD), a spectrometer (Jobin-Yvon, Horiba) and a charged coupled device (CCD).

### TEM

A FEI Titan G2 80-200 is used for the experiment of STEM-EDX mapping, equipped with Schottky field emission electron gun (working at 80 kV), a Cs probe corrector (DCOR, CEOS), and a Super-X EDX system with four detectors. Cliff-Lorimer method is applied for the quantification of specimens in the EDS map-analysis with build-in software (Esprit, Bruker).

## Additional Information

**How to cite this article**: Chen, Y. T. *et al.* Determination of critical diameters for intrinsic carrier diffusion-length of GaN nanorods with cryo-scanning near-field optical microscopy. *Sci. Rep.* **6**, 21482; doi: 10.1038/srep21482 (2016).

## Supplementary Material

Supplementary Information
