# Supplementary material for: Determination of critical diameters for intrinsic carrier diffusion-length of GaN nanorods with cryo-scanning near-field optical microscopy
Source: Sci Rep. 2016 Feb 15;6:21482. doi: 10.1038/srep21482 (PMC4753442; doi:10.1038/srep21482)
Supplement: Supplementary Information [file srep21482-s1.doc]

**Supporting Information**

Determination of critical diameters for intrinsic carrier diffusion-length of GaN nanorods with cryo- scanning near-field optical microscopy

Y.T.Chen, K.F.Karlsson, J.Birch, P.O.Holtz

**Department of Physics, Chemistry and Biology (IFM),** Linköping University,

SE-58183 Linköping, Sweden.


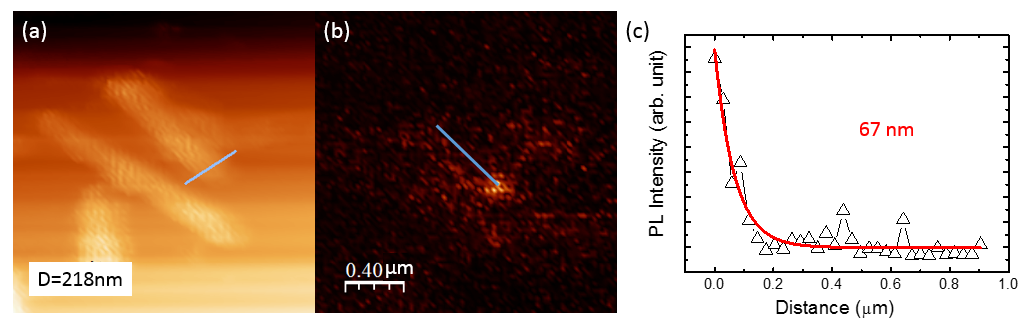

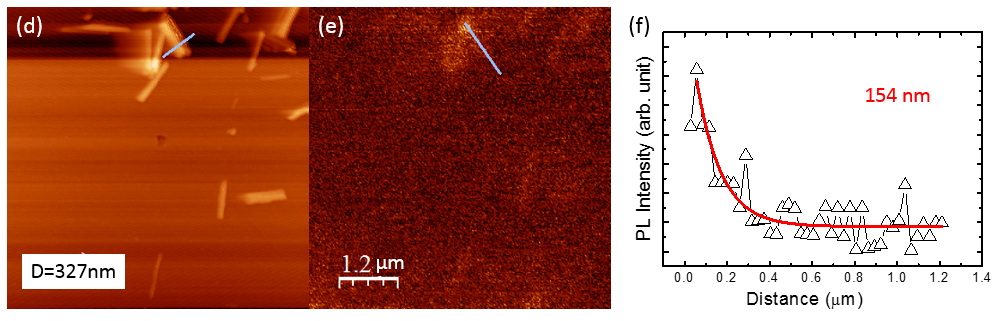

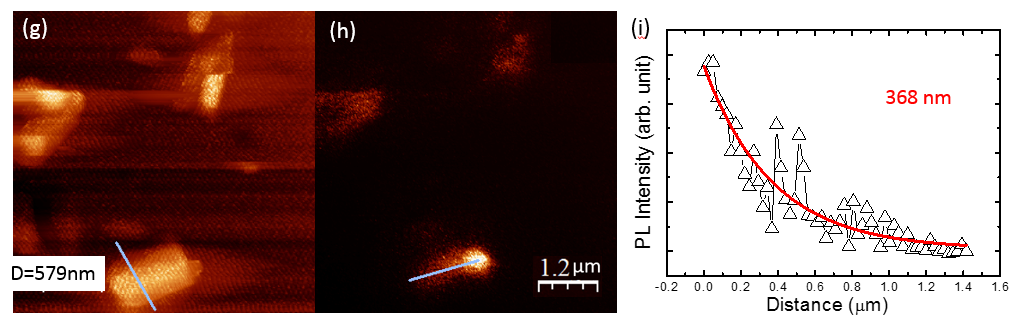

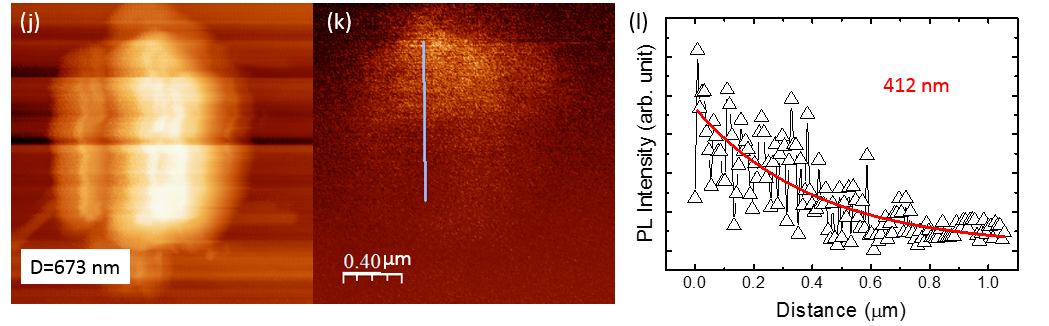


Figure S1. Detail of statistical results shown in the inset of figure 3. The height images (a)(d)(g)(j) and the related SNOM images (b)(e)(h)(k) are shown. The diffusion lengths shown in (c)(f)(i)(l), denoted in red color are calculated with the fitting of single exponential decay.
